# Dynamics associated with spontaneous differentiation of ovarian stem cells in vitro

**DOI:** 10.1186/1757-2215-7-25

**Published:** 2014-02-25

**Authors:** Seema Parte, Deepa Bhartiya, Hiren Patel, Vinita Daithankar, Anahita Chauhan, Kusum Zaveri, Indira Hinduja

**Affiliations:** 1 Stem Cell Biology Department, National Institute for Research in Reproductive Health, Mumbai 400 012, India; 2 Department of Obstetrics and Gynecology, King Edward’s Memorial Hospital, Mumbai, India; 3 Department of Obstetrics and Gynecology, Jaslok Hospital & Research Centre, Mumbai, India

**Keywords:** Ovary, Stem cells, Oogenesis, Germ cell nest, Cyclosis, Balbiani body

## Abstract

**Background:**

Recent studies suggest that ovarian germ line stem cells replenish oocyte-pool in adult stage, and challenge the central doctrine of ‘fixed germ cell pool’ in mammalian reproductive biology. Two distinct populations of spherical stem cells with high nucleo-cytoplasmic ratio have been recently identified in the adult mammalian ovary surface epithelium (OSE) including nuclear OCT-4A positive very small embryonic-like (VSELs) and cytoplasmic OCT-4 expressing ovarian germ stem cells (OGSCs). Three weeks culture of scraped OSE cells results in spontaneous differentiation of the stem cells into oocyte-like, parthenote-like, embryoid body-like structures and also embryonic stem cell-like colonies whereas epithelial cells attach and transform into a bed of mesenchymal cells. Present study was undertaken, to further characterize ovarian stem cells and to comprehend better the process of spontaneous differentiation of ovarian stem cells into oocyte-like structures in vitro.

**Methods:**

Ovarian stem cells were enriched by immunomagnetic sorting using SSEA-4 as a cell surface marker and were further characterized. Stem cells and clusters of OGSCs (reminiscent of germ cell nests in fetal ovaries), were characterized by immuno-localization for stem and germ cell specific markers and spontaneous differentiation in OSE cultures was studied by live cell imaging.

**Results:**

Differential expression of markers specific for pluripotent VSELs (nuclear OCT-4A, SSEA-4, CD133), OGSCs (cytoplasmic OCT-4) primordial germ cells (FRAGILIS, STELLA, VASA) and germ cells (DAZL, GDF-9, SCP-3) were studied. Within one week of culture, stem cells became bigger in size, developed abundant cytoplasm, differentiated into germ cells, revealed presence of Balbiani body-like structure (mitochondrial cloud) and exhibited characteristic cytoplasmic streaming.

**Conclusions:**

Presence of germ cell nests, Balbiani body-like structures and cytoplasmic streaming extensively described during fetal ovary development, are indeed well recapitulated during in vitro oogenesis in adult OSE cultures along with characteristic expression of stem/germ cell/oocyte markers. Further studies are required to assess the genetic integrity of in vitro derived oocytes before harnessing their clinical potential. Advance in our knowledge about germ cell differentiation from stem cells will enable researchers to design better in vitro strategies which in turn may have relevance to reproductive biology and regenerative medicine.

## Background

Emerging literature suggests presence of germ line stem cells in mammalian ovary, however, their ability to replenish germ cell pool in adult or postnatal stage during reproductive phase is still not acceptable within the scientific community. In a pioneering work by Bukovsky’s group [1], they suggested that ovarian surface epithelium (OSE) is a possible source of primordial follicle (PF) formation in adult stage. Professor Tilly’s group however was the first to assertively challenge the six decades old paradigm by a quantitative approach of follicular counts and showed that mouse ovary would be devoid of follicles by young adulthood [2] and thus provided evidence in support of post-natal oogenesis. Further this group demonstrated that both adult mice and human ovaries possess mitotically active germ cells that can differentiate into oocytes both in vitro and in vivo [3]. Germ-line stem cells have been reported in mice and human ovaries by several groups and have been recently reviewed elaborately [4-6].

Studies on human ovarian stem cells are relatively few in number because of scarcity of ovarian tissue for research. Bukovsky et al. [7] first demonstrated differentiation of surface epithelium of post-menopausal human ovary and development into oocytes and blastocysts in vitro. They pointed out that these stem cells generated functional oocytes in vitro irrespective of age and condition (menopausal and premature ovarian failure) unlike the conventional IVF procedure where autologous follicular oocytes are not preferred due to the genetic abnormalities harbored during aging [8]. Later Virant-Klun and her group [9-11] identified putative stem cells in ovarian sections and also in scraped ovarian surface epithelium (OSE) in post-menopausal women and those with POF which could differentiate into bonafide oocyte-like structures and parthenotes in vitro confirmed by fluorescence in situ hybridization (FISH). Our group has reported two distinct stem cell populations namely, pluripotent very small embryonic like stem cells (VSELs) with nuclear OCT-4A and slightly bigger progenitor stem cells termed as ovarian germ stem cells (OGSCs) with cytoplasmic OCT-4 in peri-menopausal women and other higher mammalian species [12] similar to our earlier results in adult human [13] and mice [14] testes. These putative stem cells differentiated spontaneously in cultures to give rise to oocyte-like structures and parthenotes [12] and expressed both pluripotent (OCT-4, OCT-4A, SSEA-4, NANOG, SOX-2, TERT and STAT-3) and germ (C-KIT, DAZL, GDF-9, VASA) cell markers. We further proposed that the OSE cells undergo epithelial-mesenchymal transition in vitro into mesenchymal fibroblasts which may act like supporting somatic granulosa cells and provide the niche (source of growth factors and cytokines) to the differentiating stem cells since developing oocytes were invariably found in close association with the mesenchymal fibroblasts [12]. Based on these findings, we have recently proposed a mechanism for possible follicular assembly in adult postnatal ovary [15]. Time lapse imaging of developing oocyte –like cells with distinctly moving cytoplasmic extensions have been reported by Bukovsky et al. [8]. A similar intimate association between developing oocyte and surrounding somatic cells has been elegantly documented by live cell imaging by Bukovsky et al. [16]. Our group has also recently reported that mouse ovarian stem cells lodged in the OSE are modulated by pregnant mare serum gonadotropin (PMSG) resulting in neo-oogenesis and postnatal follicular assembly [17]. Ovarian stem cells also respond to FSH and bFGF demonstrated by corresponding increase in expression of transcripts specific for pluripotent stem and early germ cells during organ culture of marmoset and human ovarian cortical tissue [18]. Recently our group has gathered evidence that FSH modulates ovarian stem cells through alternatively spliced variant of FSH receptor, that these stem cells undergo potential self-renewal and clonal expansion as ‘germ cell cysts’ in sheep OSE in vitro [19] and that PMSG treatment results in augmented stem cell activity, neo-oogenesis and primordial follicle assembly in mice [17].

Process of oogenesis during fetal development is accompanied by differentiation of pluripotent primordial germ cells into oogonia, proliferation of oogonia, germ cell nest formation, meiosis initiation, further differentiation of oocytes and primordial follicle assembly [20,21]. Germ cell nests or “cysts” arise by rapid incomplete mitotic divisions of the germ cells and their formation is considered to be a conserved process in vertebrates [20]. It is believed that during the nest formation, an active exchange of cellular components like mRNA and cellular organelles occurs between the developing germ cells. The germplasm, or the germ line determining RNAs become intimately associated with mitochondria and other organelles to form the Balbiani body also referred as the “mitochondrial-cloud” [21]. Balbiani bodies arise in fetal and neonatal germline nests, persist in peri-nuclear position in an asymmetric manner in resting follicles and later disperse once the growth commences in the viscous ooplasm by a process termed cytoplasmic streaming/cyclosis [22].

Despite remarkable progress made towards the quest for ovarian stem cells since the first landmark study of Johnson et al. in 2004 [2], skepticism favouring the central dogma of ‘fixed germ cell pool’ persists and the underlying controversy continues till date. This is because, specific studies refute follicular renewal in adult stage with the aid of several methods (immuno-localization, transplantation post germ cell ablation, follicular counts, lineage tracing etc.) [23-28]. Hence a consensus amidst the scientific community on this topic is still awaited.

With this backdrop of existing information, knowledge about the dynamics associated with spontaneous differentiation of adult ovarian stem cells into oocyte-like structures would further our understanding in this subject. The present study was undertaken to further characterize pluripotent ovarian stem cells lodged in the mammalian OSE using various approaches including immunomagnetic separation, RT-PCR and immuno-characterization studies. Various events associated with spontaneous differentiation of ovarian stem cells into oocyte-like structures over a period of three weeks in culture were also investigated. Further studies are required to assess the genetic integrity of in vitro derived oocytes before harnessing their clinical potential.

## Materials and methods

Human and sheep ovarian tissue have been employed for investigation and in-depth characterization of ovarian stem cells. Human OSE stem cells were characterized by immuno-localization, confocal and live cell imaging prior and post culture. SSEA-4 based immunomagnetic separation and enrichment of OSE stem cells followed by characterization of m-RNA transcripts and protein for markers of pluripotent stem cells was performed in sheep as human ovarian tissue is not readily available.

### Procurement of adult human and sheep ovarian tissue

Appropriate ethical approvals were acquired from Institutional Human and Animal Ethics Committees of King Edward’s Memorial (KEM) and Jaslok Hospital and Research Centre and National Institute for Research in Reproductive Health (NIRRH). A written informed consent was obtained from human subjects prior to surgery. Ovarian tissue collected during surgeries carried out at Gynecologic unit of KEM Hospital and Jaslok Hospital and Research Centre, Mumbai, were brought to NIRRH for further studies. Biopsies of ovarian tissue were obtained from peri-menopausal women (n = 7) with age range of 40–60 years undergoing total abdominal hysterectomy or ovariectomy due to various pathologies other than ovarian pathology, infection or malignancy as described earlier [12]. Sheep ovaries were procured from local abattoir in normal saline at ambient temperature and brought to the laboratory. Further protocol for processing of tissues was similar for both samples.

### Establishment of OSE cultures

The scraped OSE cells were maintained in culture in pre-equilibrated DMEM/F12 media supplemented with antibiotics and 20% fetal bovine serum (Invitrogen, Carlsbad, USA) in 5% CO_2_ incubator at 37°C, as described earlier [12]. The OSE isolation method followed was originally adapted from Bukovsky et al. [7] which is well documented in literature for performing similar studies. Surgical brush or scissors may also be utilized for same purpose if ovariectomy is not performed and only OSE cell brushings are retrieved from the subject [8]. Disposable culture-ware (NUNC-Part of Thermo Fisher Scientific Inc, USA) was used throughout the study. Media was partially changed on regular basis by placing the culture plates on the warm-stage of IVF workstation maintained at 37°C (K Systems; Kivex Biotech Ltd, Denmark) and the cultures were carefully monitored regularly under an inverted microscope (Eclipse TE2000-S; Nikon, Japan) with Hoffman optics.

### Enrichment of SSEA-4 positive cells

Since human ovarian tissue is scarce, detailed characterization of scraped OSE and enrichment of stem cells by immunomagnetic separation for further characterization purpose were performed using sheep ovaries. After overnight culture, single cell suspension of sheep OSE cells were collected and 1×10^8^ cells/ml were subjected to immunomagnetic based separation using SSEA-4, a cell surface pluripotent marker. Cells were incubated with EasySep® SSEA-4 Selection Cocktail on ice for 30 minutes and subsequently with EasySep® Magnet Nanoparticles (STEMCELL Technologies Inc, Labmate Asia Private Limited, India) for 20 minutes. SSEA-4 positive cells bound to EasySep® magnet were collected and used to make smears for H & E staining, confocal microscopy and also immersed in TRIZOL (Invitrogen) reagent and stored at -80°C for RNA extraction. SSEA-4 positive and negative cell fractions were recovered and suspended in DPBS. After centrifugation at 1000 g for 10 minutes at room temperature, cells were fixed in fresh 4% paraformaldehyde (pH 7.4; Sigma Aldrich, USA) for 10 min. Cell smears were made on poly-l-lysine coated slides, air dried and stored at 4°C. Both sheep and human OSE cell smears were H & E stained. H & E staining was also done on human OSE cells after 5, 7 and 21 days in culture.

### Immuno-localization studies

The initial scraped ovarian epithelial cells intermixed with VSELs and OGSCs were characterized for pluripotent, primordial germ cell, meiotic and germ cell markers (details of various antibodies are listed in Additional file 1: Table S1). In addition, the differentiation of OGSCs into oocyte-like structures at specific time points i.e. day 0, day 7 and day 21 respectively were also studied for various germ cell markers.

#### Immuno-cytochemistry

For immuno-localization of nuclear markers, the cells were permeabilized with 0.3% Triton X-100 for 10 minutes at room temperature, whereas this step was avoided while staining cells for cell surface and membrane bound markers. Non-specific epitopes were blocked by incubation at room temperature for one hour by using 20% normal serum of host species in which secondary antibody was raised, followed by overnight incubation of cell smears/culture plates with respective primary antibody at 4°C. The smears were given several washes with DPBS, and then incubated with secondary antibody, followed by avidin-biotin complex labeling (Vectastain Elite ABC system, Vector Laboratories Inc, Burlingame, USA) for 30 min each. The color reaction was carried out using 0.05% diaminobenzidine (Sigma Aldrich, St Louis, USA) and 0.05% hydrogen peroxide (Qualigens Fine Chemicals) in DPBS. The reaction was terminated after 5 minutes depending on color development by dipping the slides in tap water. The cell smears were counterstained with hematoxylin, dehydrated, cover slipped and mounted in DPX (Qualigens Fine Chemicals). The slides were examined under upright microscope (90i, Nikon) and representative fields at magnification X400 were recorded.

#### Immuno-fluorescence staining and confocal microscopy

Blocking of non-specific epitopes was carried out by incubation at room temperature for one hour with DPBS containing 3% bovine serum albumin and 0.1 mM EDTA. The cells were then incubated overnight with respective primary antibodies at 4°C, then washed with washing buffer (DPBS containing 0.5% bovine serum albumin and 0.1 mM EDTA) and later incubated with Alexafluor 488 (Molecular Probes, Invitrogen, Carlsbad, USA) labeled anti- mouse IgG, anti- goat IgG or anti- rabbit IgG (1:1,000) in wash buffer for 2 hours followed by several washes. After completion of immuno-staining protocol, the cells were counterstained for 25 seconds with 4’, 6-diamidino-2-phenylindole (DAPI), (Sigma Aldrich; 2 μg/mL) and with propidium iodide (PI, Sigma Aldrich; 0.5 μg/ml) mounted in Vecta Mount medium (Vector Laboratories Inc, USA) and examined under a laser scanning confocal fluorescent microscope (LSM 510-META; ZEISS, Germany). Images of representative fields were captured by confocal microscope at 63X magnification with optical zoom using argon laser at λ = 488 nm, blue diode laser at λ = 405 nm and DPSS laser at λ = 561 nm for observing FITC, DAPI and PI staining channels. Immuno-flourescence staining protocol of germ cell nests in whole mount mice fetal ovaries was followed as described earlier [20].

All the experiments were always conducted in triplicates and respective negative controls with omission of primary antibody were included. Primary antibody dilutions and immuno-localization protocols were optimized in the laboratory. The panels for each marker are composites prepared after examination of various representative fields to ensure accuracy of results.

### RNA extraction and RT-PCR of SSEA-4 positive & negative cell fractions

Cells obtained from both SSEA-4 positive and negative fractions were pelleted down and immersed immediately in TRIZOL (Invitrogen) reagent and stored at -80°C for RNA extraction. Post RNA extraction, the samples were treated with DNAse 1 (Qiagen) and first strand c-DNA was synthesized using Sensiscript Reverse Transcription Kit (Qiagen) according to the manufacturer’s instructions. Details of the protocol and all conditions followed were as described earlier [12].

### Documentation of germ cell growth and live cell imaging in human OSE cultures

Cellular changes were documented by live cell imaging and by capturing bright field images in Hoffmann optics on an inverted microscope (Eclipse TE2000-S). Live videos were recorded at X200 and X400 magnification with aid of Oosight™ Ultra Imaging system (CRi, Caliper Life Sciences, USA) and Image-Pro Plus image analysis software-version 5.1 (Media Cybernetics Inc, USA).

### Mitochondrial staining of germ cells

Detection of Balbiani body-like structures or the ‘mitochondrial clouds’ was performed in the live oocyte-like structures by using MitoTracker green FM dye (Life Technologies, CA, USA) and also on fixed oocyte-like structures using anti-Cytochrome c antibody (BD Biosciences, CA, USA).

MitoTracker is a chemically reactive photo-stable dye which links to thiol groups in the mitochondria and emits fluorescence once it binds and accumulates in the membrane lipids of mitochondria regardless of membrane potential. 350nM of Mito Tracker Green FM dissolved in 1 mM DMSO was added to the pre-warmed culture medium and incubated with the cells for 45–60 minutes at 37°C. Plates were covered in aluminum foil to avoid exposure to light. The dye containing medium was later replaced with pre-equilibrated original culture medium (i.e. DMEM/F12 supplemented with 20% fetal bovine serum) and the cells were observed under confocal microscope. Working concentration of the dye and incubation time used for labeling, were optimized and the final experiments were performed in replicate.

Anti-Cytochrome c antibody was used to detect the Balbiani body-like structures in fixed oocyte-like structures. For this, cultured cells were briefly fixed in chilled methanol at −20°C for 5 minutes followed by permeabilization with 0.1% Triton-X-100 for 10 minutes, followed by blocking in DPBS containing 3% bovine serum albumin and 0.1 mM EDTA prior to overnight incubation with primary antibody at 4°C. Next day, after wash with the buffer to remove unbound primary antibody, the smears were incubated with Alexafluor 488 labeled anti-mouse IgG (1:1,000, Life Technologies) in wash buffer for 2 hours. After several washes, the cells were counterstained for 25 seconds with propidium iodide (Sigma Aldrich; 0.5 μg/mL), mounted in Vecta Mount and examined under confocal microscope.

### Fluorescent in situ hybridization (FISH) of germ cells in culture

FISH was carried out using centromeric probes for X & Y chromosomes (Abbott Labs, USA). Cells fixed with 4% PFA were cytospun on a glass slide and FISH was carried out using manufacturer’s instructions. Briefly the smears were dehydrated in ascending series of ethanol followed by incubation with fluorescein isothiocyanate-labeled probes for X, Y chromosomes. Rubber cement was used to seal a cover slip on the smear. After denaturation at 75°C for 5 minutes, hybridization was carried out overnight at 37°C in a humid chamber. Next day after washing with buffer, the cells were counterstained with DAPI (Sigma; 2 mg/mL) and observed under fluorescent microscope for FISH signals under inverted microscope (TE 2000E, Nikon, Japan). Representative fields were photographed.

## Results

**Figure 1:**
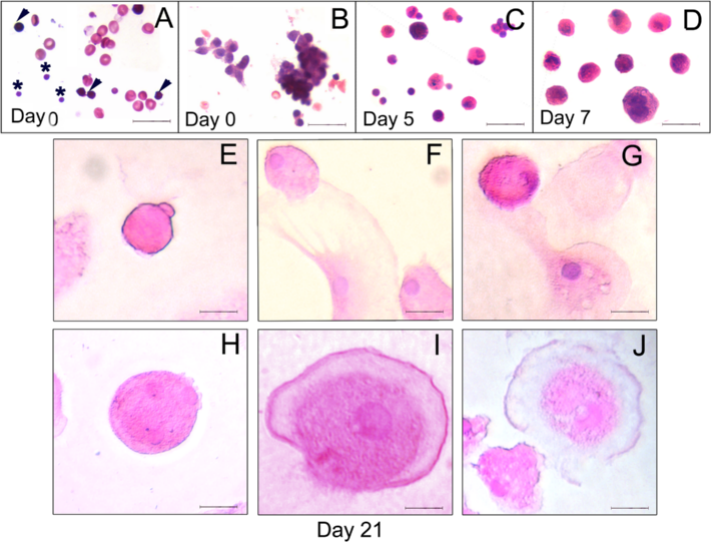
**H & E staining of human OSE cells prior and post culture. (A)** Cell smear obtained after scraping OSE comprises of two distinct populations of stem cells, viz. small sized VSELs (asterix) and slightly larger OGSCs (arrowhead) with intensely Hematoxylin stained nuclei. These stem cells are spherical in shape with high nucleo-cytoplasmic ratio **(B)** Epithelial cells with abundant cytoplasm and pale stained nuclei and RBCs are also visualized in the smears. Small clusters of cells with dark stained nuclei like the OGSCs are also observed in some fields. Similar observations were confirmed in OSE scrapings of sheep (Figure 2) **(C)** After 5 days in culture, OGSCs interspersed with few VSELs are visible. An increase in size and reduced nucleo-cytoplasmic ratio of the OGSCs is observed indicative of their growth and differentiation in vitro **(D)** After one week culture, small oocyte-like structures with abundant cytoplasm are observed **(E-J)** Larger oocyte-like structures are observed by the end of three weeks in culture in close vicinity of mesenchymal fibroblast cells. Small oocytes of 30–50 μm diameter attained a maximum size of 100 μm after three weeks of culture. Scale bar = 20 μm.

**Figure 2:**
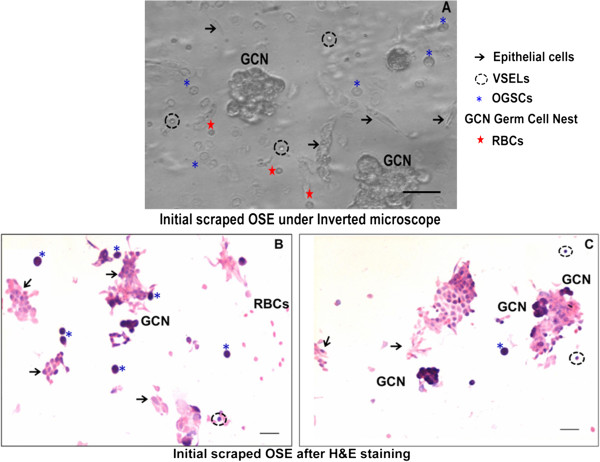
**H & E staining and observation of sheep OSE cell scrapings prior to immunomagnetic separation.** OSE cell smear obtained after scraping sheep ovaries observed under **(A)** inverted microscope and **(B, C)** after H & E staining. As evident the OSE smear comprises of epithelial cells (arrow), small sized VSELs (dotted circle), OGSCs (asterisk) germ cell nests (GCN) and red blood cells (RBCs). H & E staining shows the presence of epithelial cells with pale stained nucleus, abundant cytoplasm and pink colored RBCs which were devoid of nuclei. Stem cells of two distinct sizes with dark stained nuclei and minimal cytoplasm including VSELs and OGSCs were observed along with small clusters which were the germ cell nests. Scale bar = 20 μm.

Two distinct populations of stem cells viz. VSELs (1–3 μm in size) and OGSCs (4–7 μm in size) were identified from OSE scrapings intermixed with epithelial cells and red blood cells in both human (Figure 1) and sheep (Figure 2) which is in confirmation with our previous published report [12]. Both VSELs and OGSCs have distinct spherical shape, high nucleo-cytoplasmic ratio and stained intensely with Hematoxylin. In addition, few small clusters of OGSCs reminiscent of germ cell nests were also observed. The epithelial cells have abundant cytoplasm, elliptical shape and pale stained nuclei.

Upon culture in high glucose containing DMEM/F12 media, VSELs and the progenitor OGSCs undergo proliferation and further differentiation into oocyte-like structures (Figure 1), as described in earlier published results [12,15]. The stem cells underwent growth as evident by Day 5 (Figure 1C), with increase in size accompanied by decreased nucleo-cytoplasmic ratio. They differentiated into smaller oocyte-like structures by one week in vitro (Figure 1D). By Day 21, larger oocyte-like structures developed which exhibited increased proportion of cytoplasm. The growing oocyte-like structures were invariably observed in close association with fibroblasts, resembling the supporting granulosa-like cells possibly formed by epithelial to mesenchymal transition (EMT) of the ovarian epithelial cells in culture (Figure 1E-J).

### Immuno-localization studies in human OSE cells prior and post culture

**Figure 3:**
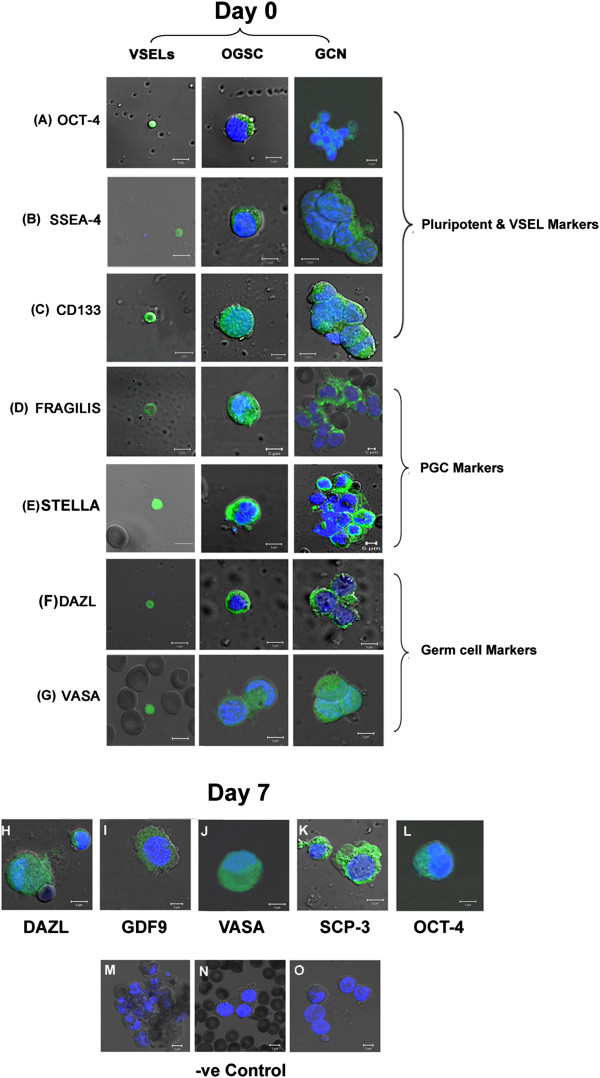
**Characterization of ovarian stem cells on Day 0 and Day 7 by confocal imaging.** Pluripotent markers **(A)** OCT-4 **(B)** SSEA-4 **(C)** CD133; PGC markers **(D)** FRAGILIS **(E)** STELLA and germ cell markers **(F)** DAZL **(G)** VASA were immuno-localized to the VSELs and OGSCs at Day0. The oocyte-like structures observed on Day7 post culture expressed germ cell markers **(H)** DAZL, **(I)** GDF-9 **(J)** VASA **(K)** SCP-3 and **(L)** OCT-4. DAPI is used as a nuclear counter stain. **(M-O)** Mouse, rabbit and goat negative controls with omission of primary antibody were immuno-stained similarly. Scale bar = 5 μm. Merged image of DAPI, FITC and DIC (Differential Interference contrast).

Pluripotent marker OCT-4 was prominently localized to the nucleus of VSELs and in the cytoplasm of the OGSCs (Figure 3A). SSEA-4 was localized to the cell surface of VSELs and minimally in the cytoplasm of OGSCs (Figure 3B). Similarly distinct staining for CD133 was observed in the VSELs and was localized in the OGSCs (Figure 3C). Cell surface and nucleus specific primordial germ cell markers FRAGILIS and STELLA were localized to the VSELs respectively and in the cytoplasm of the OGSCs (Figure 3D and E). Germ cell specific markers DAZL and VASA were found to be expressed in the nuclei and cytoplasm of VSELs respectively and in the cytoplasm of progenitor OGSCs on Day 0 (Figure 3F, G). The oocyte-like structures harvested on Day 7 and 21 of culture expressed all the germ cell specific markers (DAZL, GDF-9, VASA, SCP-3, OCT-4) in the cytoplasm (Figure 3H-L, Additional file 2: Figure S1 and Additional file 3: Figure S2). Interestingly the germ cell nest/cyst-like structures stained positive for various pluripotent and germ cell markers like OCT-4B, SSEA-4, CD133, FRAGILIS, STELLA, DAZL and VASA (Figure 3). The expression pattern of various markers studied for characterization of VSELs and OGSCs prior to and post culture are mentioned in Table 1. In situ hybridization studies using OCT-4 specific oligoprobe on sheep OSE cells confirmed the presence of nuclear OCT-4 positive stem cells (Additional file 4: Figure S3) in agreement with our earlier results in testis [13].

**Table 1:** Expression pattern of various markers in stem cells and developing oocytes during culture

**Markers**	**VSELs**	**OGSCs**	**Germ cell nests/cysts**	**Oocyte-like structures (1 week culture)**	**Oocyte-like structures (3 weeks culture)**
OCT-4A	N	-	-	-	-
OCT-4B	-	C	C	C	C
SSEA-4	CS	C	C	C	C
CD133	CS	C	C	ND	ND
FRAGILIS	CS	C	C	ND	ND
STELLA	N	C	C	ND	ND
DAZL	N	C	C	C	C
VASA	C	C	C	C	C
SCP-3	-	-	-	C	C
GDF-9	-	-	-	C	C

N: nuclear; CS: cell surface; C: cytoplasmic; ND: not done; -: not detected.

### Enrichment of SSEA-4 possitive cells from sheep OSE

**Figure 4:**
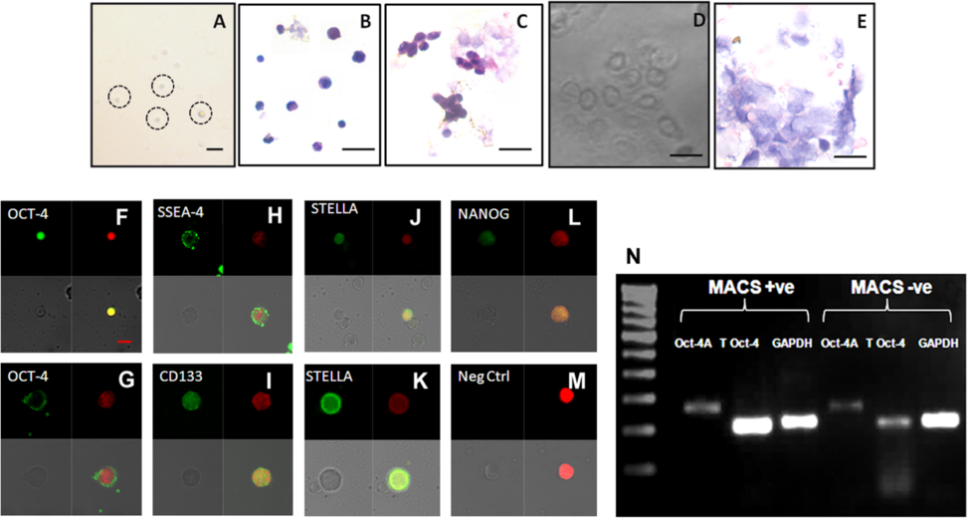
**Characterization of sheep OSE cells post SSEA-4 based immunomagnetic cell separation.** SSEA-4 positive separated cells comprised of spherical stem cells of two distinct sizes and high nucleo-cytoplasmic ratio were observed in **(A)** Bright field and after **(B)** H & E staining. **(C)** Germ cell nests were also observed with few attached epithelial cells. The cells that did not get selected mainly comprised of **(D, E)** epithelial cells seen under bright field and after H & E staining respectively. The sorted cells expressed both **(F, G)** nuclear and cytoplasmic OCT-4, **(H)** cell surface SSEA-4 and **(I)** CD133, **(J, K)** STELLA in both stem cells, **(L)** NANOG, by confocal microscopy. **(M)** Negative control was employed by omission of primary antibody. (F-M is a composite of four panels representing fluorescent channels, bright field and merged images of both by confocal microscopy). **(N)** RT-PCR for Oct-4A, Oct-4 and Gapdh in SSEA-4 positive sorted fractions showed bands of expected size (290, 225 and 232 base pairs respectively using PCR conditions and primers described earlier, [12]). Low intensity bands were also detected in the negative fraction possibly because of the germ cell nests which were observed in both the fractions. Scale bar = 10 μm in A, 20 μm in B-E and 5 μm in F-M.

Two stem cell populations and germ cell nests/cysts were identified in whole OSE cell suspension of sheep and after H & E staining under a microscope (Figure 2) and further enriched using SSEA-4 (a pluripotent stem cell surface marker) antibody based immunomagnetic cell separation method. H & E staining of SSEA-4 positive cell fraction revealed spherical stem cells of two distinct sizes and possessed a high nucleo-cytoplasmic ratio. Germ cell nests were also observed with few attached epithelial cells (as a result of mechanical disaggregation of OSE). The cells that did not get selected (SSEA-4 negative cell fraction) mainly comprised of epithelial cells. Few germ cell nest-like structures remained intact and tiny spherical cells with high nucleo-cytoplasmic ratio were also observed occasionally in SSEA-4 negative cell fraction (Figure 4). This technique enabled to enrich and identify the pluripotent stem cell population in OSE cell scrapings which, in our generic OSE cultures differentiated into germ cells and other cell types wherein these differentiated cells in turn were originally not observed within the OSE layer.

### Characterization of SSEA-4 expressing cells from sheep OSE

Immunomagnetically sorted and enriched stem cells were further confirmed for expression of pluripotent stem & VSELs specific markers by confocal microscopy. The SSEA-4 possitive cells expressed both nuclear and cytoplasmic OCT-4, NANOG, SSEA-4, CD133 and STELLA by confocal microscopy. Respective negative controls were employed by omission of primary antibody (Figure 4). Besides, expression of m-RNA transcripts specific for VSELs and OGSCs were confirmed by semi-quantitative RT-PCR. m-RNA transcripts specific for Oct-4A, total Oct-4 and house-keeping gene Gapdh were present in sorted SSEA-4 positive cell fraction and bands of expected size (290, 225 and 232 base pairs respectively using PCR conditions and primers described earlier [12] were observed (Figure 4). However, low intensity bands were also detected in the SSEA-4 negative cell fraction due to technical reason more than a biological one. All experiments were conducted in triplicates from three independent experiments with respective RT and PCR no template controls.

### Cytoplasmic streaming movement in human OSE cultures

**Figure 5:**
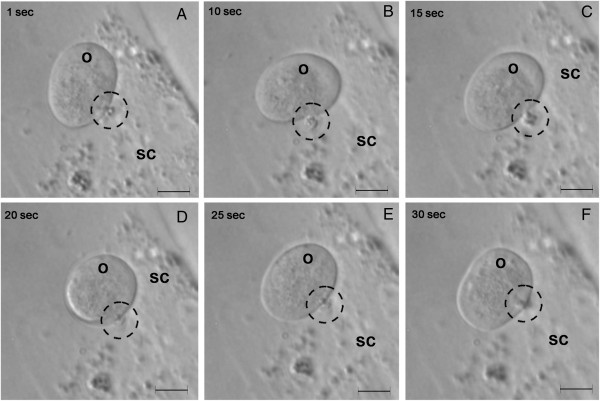
**Cross-talk between oocyte-like structure (O) and somatic mesenchymal fibroblast cell (SC) exhibiting cytoplasmic streaming movement during development in culture.** Live cell imaging of OSE cultures at regular intervals demonstrates that the cytoplasmic fragment pinched off (dotted circle) from the SC is being engulfed by the O which is also undergoing cytoplasmic movement at the same time. Simultaneously note the change in shape of O at **(A)** 1 second **(B)** 10 seconds **(C)** 15 seconds **(D)** 20 seconds **(E)** 25 seconds and **(F)** 30 seconds. Scale bar = 20 μm.

**Figure 6:**
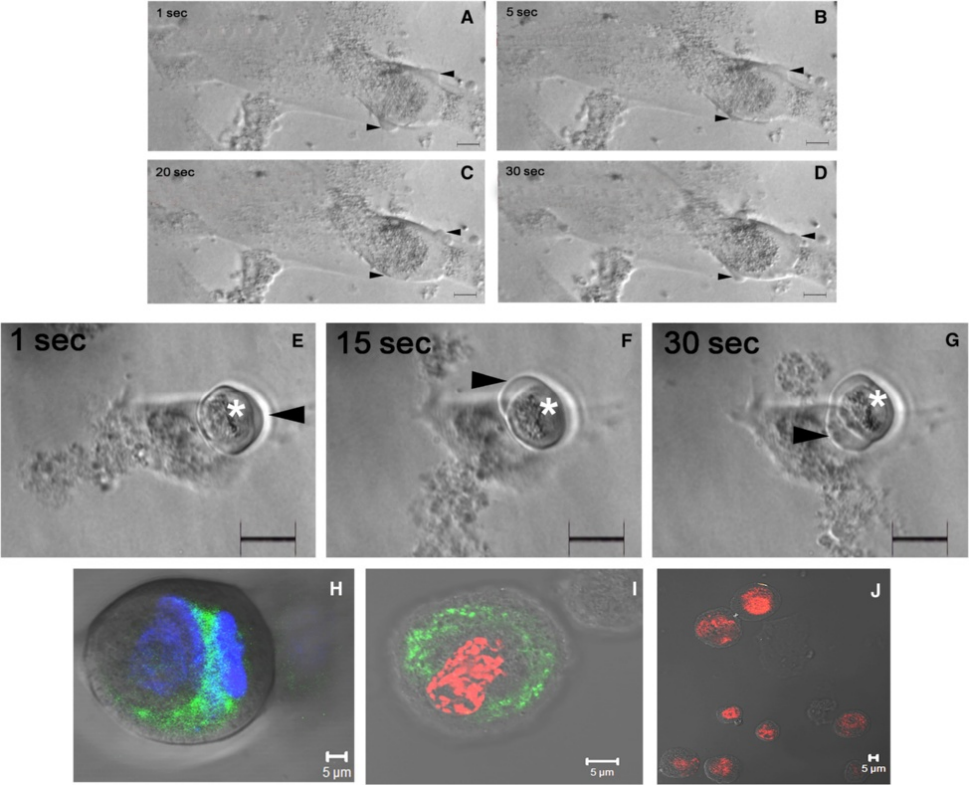
**Live-cell imaging of cytoplasmic streaming and mitochondrial cloud (MitoTracker staining and Cytochrome c localization) in somatic fibroblasts and oocyte-like structures.** The somatic mesenchymal fibroblast cells (top panel) show subtle change in morphology (arrowhead) due to the dynamic cytoplasmic streaming movement or blebbing typically observed at cell periphery during its development in culture at different time intervals at **(A)** 1 second **(B)** 5 seconds **(C)** 20 seconds and **(D)** 30 seconds from initiation of observation. The central region of small oocyte-like structure (middle panel) shows concentration of electron dense material (white asterisk). Note the gradual change in shape near its periphery (arrowhead) due to the subtle cytoplasmic movement imaged at **(E)** 1 second **(F)** 15 seconds **(G)** 30 seconds. Dense ring of mitochondrial cloud (lower panel) was visible within the oocyte-like structure in live cultures by **(H)** MitoTracker green FM staining and **(I)** immuno-localization of Cytochrome c **(J)** Mouse negative control was maintained by omission of primary antibody. Scale bar = 20 μm in A-G and 5 μm in **H-J**.

Cytoplasmic streaming movement was observed in the OSE cultures by end of one week, which continued till termination of cultures. Both somatic mesenchymal fibroblasts acting like supporting granulosa cells and the oocyte-like structures showed distinct cytoplasmic streaming movement (Figures 5 and 6). The somatic cells were attached to the bottom of culture dish and occasionally showed subtle and/or prominent blebbing on their surface (Figure 6, Additional file 5: Video S1). Small, dense cytoplasmic content appeared to pinch off from surface of the surrounding somatic cell (Additional file 5: Video S1 and Additional file 6: Video S2) and were taken up by the developing germ cells (Figure 5) as also observed by live cell imaging. Spherical oocyte-like structures with electron dense central portion had clear cytoplasm at periphery (Figures 5 and 6) and exhibited characteristic cytoplasmic streaming or rotational movement during the initial stage of culture. This movement was uniform, continuous (either in clock or anti-clockwise direction) and lasted for few minutes only. Continuous observation of cultures was not possible as they had to be maintained for a further period of three weeks. The observations were confirmed by monitoring cultures for only short durations at regular intervals of time accompanied by live cell imaging and documentation of cytoplasmic streaming movement every other day. Dynamic changes in the ooplasm during its growth in vitro were evident by the gliding movement of clear cytoplasm which subsequently resulted in changes in morphology of the germ cells developing in culture (Figure 5, 6, Additional file 5: Video S1 and Additional file 6: Video S2).

### Mitochondrial staining in spontaneously differentiated oocytes

Well defined aggregates of mitochondrial clouds resembling Balbiani body-like structures characteristic of oocytes were evident in the perinuclear region of small oocyte-like structures in OSE cultures. Mitochondrial clouds were detected by MitoTracker green FM dye staining and immuno-localization of Cytochrome c to characterize the oocyte-like structures derived from human OSE stem cell cultures (Figure 6).

### Characterization of germ cell cysts/nests & FISH analysis

Germ cell nests were immuno-stained for OCT-4, SSEA-4 and VASA and visualized by confocal z-stack imaging. Cytoplasmic continuity between adjacent cells was clearly evident in the germ cell nests isolated from both human and sheep ovary, similar to those observed in fetal mice ovaries used as positive control (Additional file 7: Figure S4). Fluorescent in situ hybridization (FISH) for X & Y chromosomes revealed presence of only X chromosome and appeared to be in different stages of maturation i.e. primary and secondary oocyte (Additional file 7: Figure S4).

## Discussion

Ovarian stem cells lodged in the surface epithelium include the pluripotent very small embryonic-like stem cells (VSELs) and the immediate descendants or ‘progenitors’ termed ovarian germ stem cells (OGSCs) [12,15]. Present study demonstrates that the OSE cell scrapings in culture undergo dynamic changes i.e. the stem cells spontaneously differentiate into germ cells, form ‘germ cell nests/cysts’, become bigger in size, develop abundant cytoplasm, differentiate into small and later larger oocyte-like structures, reveal presence of mitochondrial cloud i.e. Balbiani body-like structures within the oocytes and also exhibit characteristic cytoplasmic streaming. This is associated with precise expression of markers specific for pluripotent VSELs (nuclear OCT-4, SSEA-4, CD133), progenitors OGSCs (cytoplasmic OCT-4), primordial germ cells (FRAGILIS, STELLA, VASA) and oocyte-like structures (DAZL, GDF-9, SCP-3). Both the stem cell populations showed the presence of Oct-4 m-RNA by in situ localization in sheep OSE cells similar to earlier reported results in human testis [13]. Stem cells in the OSE were further enriched by immunomagnetic sorting using pluripotent cell surface SSEA-4 and their pluripotent nature was confirmed at both protein (nuclear and cytoplasmic OCT-4, NANOG, SSEA-4, CD133 and STELLA) and m-RNA transcript (Oct-4A, total Oct-4) levels. Similar report of SSEA-4 sorted OSE stem cells have been reported by Virant Klun’s group [29].

We admit that further studies are required to precisely identify whether the VSELs or OGSCs undergo differentiation into oocyte-like structures since the initial population of OSE cells is very generic in nature. Pluripotent stem and germ cell markers like Oct-4A, Oct-4, Nanog, Sox-2, Tert, Stat-3 have been reported in ovarian cortex and isolated OSE [12]. Similarly, up-regulation of VSELs (Oct-4A, Nanog), OGSCs (Oct-4) and germ cell markers (c-Kit and Vasa) were evident even during culture of human and marmoset ovarian cortical tissue (comprising the OSE) in presence of FSH and bFGF [18]. We have also recently reported increase in relative mRNA expression of both VSELs (Oct-4A) and OGSCs (Oct-4) specific transcripts during sheep OSE culture [19]. VSELs are relatively quiescent and we do not expect large increase in numbers of VSELs. VSELs undergo asymmetric cell division, self-renew and give rise to actively dividing OGSCs which is reflected by relatively larger increase in Oct-4 compared to Oct-4A transcripts [18,19]. May be we need to culture a pure population of VSELs from a GFP mice with wild-type OSE cells to unravel their differentiation potential.

After the first report on the presence of VSELs in mice by Ratajczak’s group [30], their existence in human blood, bone marrow, most adult tissues including ovary and testis has been successfully demonstrated [31] in subsequent years,. However in a recent study led by Professor Weissman at Stanford University School of Medicine, the very existence of VSELs in the mouse bone marrow and blood was questioned. The group was unsuccessful in replicating the original protocols employed for isolating VSELs and consequently failed to find VSELs in mouse bone marrow that could differentiate into hematopoietic cells [32]. The technical nuances which led to the negative results have been elaborately discussed by Ratajczak’s group recently [33].

Two groups [34,35] have reviewed the appearance of various markers in relation to germ cell development in mice and human fetal ovary. VSELs are possibly the pluripotent primordial germ cells that persist into adulthood [36]. In agreement with this concept, we observed both pluripotent and PGCs specific markers in the stem cells isolated from the OSE. Two recent papers from Tilly’s group [37,38] demonstrate the presence of a specific gene signature in the oogonial stem cells and that they respond to BMP similar to primordial germ cells. VSELs express nuclear OCT-4, cell surface CD133 and SSEA-4 whereas the OGSCs exhibited cytoplasmic OCT-4. PGC specific markers FRAGILIS and STELLA were observed in the VSELs, OGSCs and in germ cell clusters. Both DAZL and VASA reported to appear in migratory PGCs or those colonizing the gonads during development [34] were also found to be present in the VSELs. The cells in culture possibly undergo meiosis is suggested by presence of SCP-3 (a meiosis-specific marker) [39] and GDF-9 (a post-meiotic marker) [40] and FISH results. Cytoplasmic localization of meiotic marker SCP-3 in the in vitro derived oocyte-like structures could be due to the newly synthesized protein eventually destined to localize to nucleus, as observed and explained in earlier studies performed by Danner et al. [41]. Vasa, Scp-3 and Zp transcripts have also been detected in in vitro derived oocytes from OSE cultures [9,10].

Presence of germ cell nests/cysts, a characteristic feature of developing germ cells/oogonia were observed in the initial scraped OSE smears along with the VSELs and OGSCs, and also after immunomagnetic sorting. Possibly the VSELs undergo asymmetric cell division to give rise to OGSCs which in turn divide rapidly to form germ cell nests and upon further differentiation result in oocyte-like structures. Confocal z-stack imaging for OCT-4, SSEA-4 and VASA markers showed cytoplasmic continuity between germ cells similar to the germ cell nests observed within mice fetal ovaries. These nests arise due to rapid cell division and incomplete cytokinesis and have been reported in fetal ovaries and have been implicated to help in (i) synchronizing development of germ cells prior to meiosis, (ii) sharing of genetic material through intercellular bridges and (iii) mRNAs, mitochondria and ribosomes in the developing oocyte from surrounding nurse cells [28,42]. Similar clusters of 50–200 cells (distinct from follicles) with large nuclei that intensely stained with Haematoxylin and positive for Oct-4, MVH, SSEA-1, SCF-R and meiotic markers SCP-3 & DMC-1 have also been reported earlier in Oct-4 EGFP transgenic mice ovaries [43]. The detection of germ cell nests reported in adult mammalian ovaries in the present study is in contradiction to the failure to detect similar structures in mouse ovary [28] possibly due to technical reasons. Formation of germ cell cysts/nests from the stem cells in adult mammalian ovary will be a rare event and is expected to be stage specific. We have reported similar stem cell renewal resulting in germ cell cysts formation within 15 hours of sheep OSE culture. OCT-4 and SSEA-4 expressing germ cell cysts have been reported in adult sheep OSE cultures treated with FSH [19]. Also these cysts can be detected in OSE smears prepared from mouse ovary treated with PMSG [17,44] which results in increased neo-oogenesis and primordial follicle assembly [17]. We have recently reported presence of rare germ cell cysts/nests in adult human, sheep and mice ovary and testis [44]. These results offer strong support to the concept that oogenesis and primordial follicle assembly occurs in adult mammalian ovaries as suggested earlier by Bukovsky et al. [7].

In OSE cultures, epithelial cells provide a conducive niche (source of growth factors) for differentiating germ cells into oocyte-like structures. The developing oocyte (largest cell in the body) has very few organelles [45] and heavily depends on the surrounding granulosa cells for nutrients as well as organelles [46]. In the present study, a dynamic cytoplasmic streaming was observed between the somatic, germ cells and oocyte-like structures evidently associated with the differentiation and maturation of ovarian stem cells into oocyte-like structures in vitro. Cytoplasmic streaming is known to be associated with various physiological processes [47] and particularly it has been reported as a phenomenon accompanying oocyte maturation and acquisition of developmental competence [48]. Oocytes are highly polarized with respect to distribution of mRNA and proteins in the gelatinous ooplasm. Recently mouse oocytes have been reported to undergo distinct cytoplasmic streaming while completing meiosis II which is a highly asymmetrical division expelling a small polar body [49]. Verlhac [50] discussed whether such cytoplasmic streaming is also associated with the first polar body expulsion in mouse oocytes. The cytoplasmic streaming described in mouse oocytes [49] is limited by a zona pellucida and looks different compared to the movement reported in the present study where the zona pellucida restricting the oocyte at periphery is lacking in most of the developing germ cells in OSE cultures.

Oocyte-like structures that appear after spontaneous differentiation were also found to exhibit the presence of mitochondrial clouds (Balbiani-body like structures) in the peri-nuclear region in agreement with earlier report [16] which suggested that it is possibly transferred from the surrounding somatic granulosa cells. Cox and Spradling [51] have earlier reported that the somatic follicular cells in drosophila ovary are the source of virtually all the mitochondria which enter the ooplasm through interconnected ring canal at the germ cell nest stage. Results of the present study are also in agreement with Albamonte et al. [52] who have localized germ cell specific VASA in perinuclear position of ooplasm corresponding to the location of Balbiani body within a human PF oocyte. Balbiani body was recently reported in neonatal mice by Pepling’s group [53] and was associated with germline cysts and oocytes of primordial follicles which later disperse as the PF begins to grow.

To conclude, three major hallmark features of germ cell development: germ cell cysts/nest, mitochondrial aggregates (Balbiani body-like structures) and cytoplasmic streaming (cyclosis) were observed during spontaneous differentiation of stem cells into oocyte-like structures. The stem cells in a peri-menopausal ovary (devoid of any follicles/oocytes) also revealed a potential to differentiate into oocytes in vitro. Then why does menopause occur in females - demarcating the end of reproductive phase? Available literature suggests that with advancing age, it is the somatic niche where the stem cells reside gets compromised resulting in menopause [5,12] although the stem cells retain the potential to undergo oogenesis. Culture conditions facilitate the persisting stem cells in vitro to undergo spontaneous differentiation into oocyte-like structures possibly because they get exposed to enriched culture medium (improved niche) and as suggested earlier probably the inhibitory factors that may exist in vivo in menopausal ovary are overcome in vitro [54]. More studies in this direction should be undertaken which will further our understanding about the process of neo-oogenesis and follicle assembly in adult mammalian ovary and could change the approach to treat infertility in near future.

## Additional files

### *In situ* hybridization (ISH) study

Oct-4 mRNA expression was studied in sheep OSE cells using non-radioactive Digoxigenin based alkaline phosphatase system by *in situ* hybridization (Roche Diagnostics, Germany) technique. All precautions to prevent RNA degradation were taken during the experiment. Aminosilane coated glass slides were used for making sheep OSE cell smears. Cells were fixed in 2% paraformaldehyde in DPBS (Invitrogen) prepared using 0.1% DEPC treated water for 15-20 mins, rinsed twice with DPBS, air dried and stored at 4°C until use. Oligo probes and methodology used for ISH were same as we described earlier [13], (antisense) CGCTTTCTCTTTCGGGCCTGCACGAGGGTTTCTGC and (sense) GCAGAAACCCTCGTGCAGGCCCGAAAGAGAAAGCG. Digoxigenin labeling of oligo probes was performed as per the manufacturer’s instructions for 3′ tailing kit. OSE cell smears were brought to room temperature, hydrated in 0.1M PBS (pH 7.0) and refixed for 10 mins followed by wash in 0.1M PBS. Smears were further incubated with 2X sodium saline citrate (SSC) freshly prepared from a 20X stock solution (0.15 M sodium chloride and 0.015 M sodium citrate, pH 7) for 15 mins at room temperature. Smears were further incubated at 42°C for 2 hrs with pre-hybridization cocktail (50% formamide, 4X SSC, 5X Denhardt’s solution, 0.25% yeast tRNA, 0.5% sheared salmon sperm DNA, and 10% dextran sulphate) in a humid chamber. The cells were further hybridized overnight at 42°C with Digoxigenin labeled oligo probe diluted in the pre-hybridization mix at a concentration of 5 pmol/μl in a humid chamber. Next day, excess un-bound oligoprobe was removed with varying concentrations of SSC containing 0.1% Tween-20 (4X SSC, 10 mins twice; 2X SSC, 5min twice; 1X SSC, 5 min once) followed by incubation with blocking solution (2% NGS, 0.1% Triton X-100 in 0.1M Tris-HCl buffer; pH 7.5) for 2 hrs. Later the cells were incubated with alkaline phosphatase-conjugated anti-Digoxigenin antibody diluted (1:500) prepared in blocking solution overnight at 4°C. Cell smears were rinsed in 0.1 M Tris-HCl (pH 7.5) for 10-15 mins and equilibrated in 0.1 M Tris-HCl (pH 9.5) for 30 mins. Detection was performed using solution comprising of nitroblue-tetrazolium (NBT) and 5-bromo-4-chloro-2-indoyl phosphate (BCIP) containing 0.2% levamisole prepared in 0.1 M Tris-HCl (pH 9.5) at RT. Reaction was stopped by adding stop solution comprising of Tris-HCl and 10mM EDTA (pH 8.0) followed by dipping slides in distilled water and final mounting in 90% glycerol. Cell smears were viewed and representative fields were photographed using 90i bright-field microscope (Nikon, Japan). Sense probe was used as a negative control.

## Abbreviations

CD 133: Cluster of Differentiation 133; DAPI: 4’,6-diamidino-2-phenylindole; DAZL: Deleted in azoospermia-like; DNA: Deoxy ribonucleic acid; EMT: Epithelial mesenchymal transition; ESCs: Embryonic stem cells; FISH: Fluorescence in situ hybridization; GDF-9: Growth and differentiation factor; Oct-4: Octamer binding protein 4; OGSCs: Ovarian germ stem cells; OSE: Ovarian surface epithelium; OSC: Oogonial stem cells; PGC: Primordial germ cells; POF: Premature ovarian failure; RNA: Ribonucleic acid; SCP-3: Synaptonemal complex protein 3; SSEA-4: Stage-specific embryonic antigen-4; VSELs: Very small embryonic-like stem cells.

## Competing interests

The authors declare that they have no competing interests.

## Authors’ contributions

SP was involved in sample collection, culture and experimental work, data analysis and scientific inputs during manuscript drafting and preparation. DB was responsible for conceptualizing the contents of the research study, planning experiments, data interpretation, providing scientific inputs, critical drafting, reviewing and final approval of manuscript. HP and VD were actively involved in characterization studies and reviewing the manuscript. AC, IH and KZ were responsible for providing human ovarian tissue samples and critically reviewing the manuscript. All authors read and approved the final manuscript.

## Supplementary Material

Additional file 1: Table S1 Details of markers used in the study to characterize pluripotent stem cells and differentiated germ cells. Click here for file

Additional file 2: Figure S1 Immuno-characterization of ovarian stem cells on Day 0 and Day 7. Click here for file

Additional file 3: Figure S2 Immuno-characterization of germ cells in OSE cultures after three weeks. Click here for file

Additional file 4: Figure S3 Localization of Oct-4 mRNA in sheep ovarian stem cells. Click here for file

Additional file 5: Video S1 Characteristic cytoplasmic streaming movement within oocyte-like structures and fibroblasts. Click here for file

Additional file 6: Video S2 A larger oocyte-like structure showing prominent cytoplasmic streaming. Click here for file

Additional file 7: Figure S4 Confocal z-stack composites of germ cell nests in OSE smears. Click here for file
