# Supplementary material for: Dynamics associated with spontaneous differentiation of ovarian stem cells in vitro
Source: J Ovarian Res. 2014 Feb 25;7:25. doi: 10.1186/1757-2215-7-25 (PMC4234975; doi:10.1186/1757-2215-7-25)
Supplement: Additional file 1: Table S1 — Details of markers used in the study to characterize pluripotent stem cells and differentiated germ cells. [file 1757-2215-7-25-S1.docx]

**Supplementary Section 2**

**Supplementary Table: Details of Markers used in the Study to Characterize Pluripotent Stem Cells and Differentiated Germ Cells**

| **Antibody & Dilution** | **Source & Catalog Number** | **Functional Significance** | **Staining Pattern** | **Antibody used in other studies for similar characteriz-ation** | **Reference** |
| --- | --- | --- | --- | --- | --- |
| OCT-4  (1/100) | Abcam, UK  ab19857 | An octamer binding nuclear transcription factor of the POU family, with 360 amino acids used to define pluripotent state of a stem cell, well studied in embryonic stem cell (ES) and carcinoma stem cells. It is a germ-line specific maternally expressed factor expressed by PGCs and germ cells during embryonic development. It is critical for early embryogenesis and for ES cell pluripotency. Also known to be expressed in VSELs and tissue-specific progenitors. | Oct-4A isoform is nuclear in pluripotent stem cells  Oct-4B isoform is localized in cytoplasm of progenitors viz. OGSCs and GCN | Parte et al 2011[12] | [12,13, 55-57] |
| SSEA-4  (1/50) | Millipore, USA  MAB4304 | SSEA-4, a glycolipid carbohydrate epitope that is expressed upon the surface of human embryonic- carcinoma, germ and ES cells. Identiﬁes adult mesenchymal stem cell population. Also known to be expressed in VSELs and tissue-specific progenitors. | Cell surface in pluripotent stem cells and minimally cytoplasmic in progenitors viz. OGSCs and GCN | Kumar et al 2009,[58] Parte et al 2011[12] | [5, 14, 59-61] |
| CD 133  (1/50) | Millipore, USA MAB4399 | A 115-120 kDa member of Interferon-inducible pentaspan trans-membrane domain glycoprotein, marks the onset of germ cell competence, also expressed in brain tumours, a hematopoietic and neuronal stem cell marker | Cell surface in pluripotent stem cells and cytoplasmic in progenitors viz. OGSCs and GCN | personal observation | [49- 52 62-65] |
| FRAGILIS  (1/50) | Abcam, UK  ab74699 | An interferon inducible gene coding for trans-membrane protein is the first gene implicated in acquisition of germ cell competence, expressed by cells in the primitive streak during germ line development | Cell surface in pluripotent stem cells and cytoplasmic in progenitors viz. OGSCs and GCN | Bhartiya et al 2011[66] | [67-69 53- 55] |
| STELLA  (1/50) | Millipore, USA  MAB4388 | Novel gene specifically expressed in PGCs, oocytes, pre-implantation embryos and pluripotent cells, a maternal factor essential for maintenance of methylation involved in epigenetic reprogramming after fertilization, protects DNA methylation state of several imprinted loci and epigenetic asymmetry. Stella gene encodes a protein with a SAP-like domain and splicing factor motif-like structure, suggesting possible roles in chromosomal organization and RNA processing | Nuclear in pluripotent stem cells and cytoplasmic in progenitors viz. OGSCs and GCN | personal observation | [70-72] |
| DAZL  (1/200) | Abcam, UK  ab34139 | It is germ-cell-specific-RNA binding protein essentially crucial for translational initiation during germ cell development, differentiation and functional maturation of PGC, expressed in prenatal and postnatal germ cells of males and females. Protein encoded by this gene is localized to the nucleus and cytoplasm of fetal gonocytes and to the cytoplasm of developing oocytes. Transcripts are present in late stages during oocyte maturation, ES cells, ICM and TE of blastocyst. | Nuclear in VSELs and cytoplasmic in progenitors viz. OGSCs, GCN and oocytes | Parte et al 2011[12] | [12,14]  73,74] |
| GDF-9  (1/50) | Abcam, UK  ab38544 | It is a transforming growth factor-beta family member synthesized by ovarian somatic cells, has a critical role in granulosa and [theca cell](http://en.wikipedia.org/wiki/Theca_cell) growth, as well as in differentiation and maturation of the oocyte, therefore has a significant role in [fertility](http://en.wikipedia.org/wiki/Fertility). GDF-9 generates a signal via a cell surface which is the [bone morphogenetic protein](http://en.wikipedia.org/wiki/Bone_morphogenetic_protein) type II receptor | Cytoplasmic in oocytes | Parte et al 2011[12] | [14, 75-78] |
| VASA  (1/100) | R & D Systems, USA  AF2030 | Member of the germ-line–specific DEAD-box RNA helicase, maternal-effect gene required for germ-cell specification, and is required for promoting translation of at least two known mRNAs- nanos and gurken | Cytoplasmic in VSELs and cytoplasmic in progenitors viz. OGSCs, GCN and oocytes | Parte et al 2011[12] | [79-81] |
| SCP-3  (1/50) | Abcam, UK  Ab15093 | Main determinant of axial element assembly and required for attachment of this structure to meiotic chromosomes | Cytoplasmic in *in-vitro* derived oocytes | Bhartiya et al 2012[17] | [5,12, 82] |
| Cytochrome- C  (1/500) | BD Biosciences, USA  556432 | Cyt C  is a highly conserved heme protein, an essential component of the electron transport chain and is also involved in initiation of apoptosis | Mitochondrial inter-membrane space under normal physiological conditions | Basu et al 2008 [83] | [84-86] |

**References for Supplementary Section**

1. Scholer HR, Ruppert S, Suzuki N, Chowdhury K and Gruss P: **New type of POU domain in germ line-specific protein Oct-4.** *Nature* 1990, **344:**435-439.
2. Cauffman G, Liebaers I, Van Steirteghem A and Van de Velde H: **POU5F1 isoforms show different expression patterns in human embryonic stem cells and preimplantation embryos.** *Stem Cells* 2006, **24:** 2685-2691.
3. Lee J, Kim HK, Rho JY, Han YM and Kim J: **The human OCT-4 isoforms differ in their ability to confer self-renewal.** *J Biol Chem* 2006, **281** 33554-33565.
4. Kumar N, I Hinduja, P Nagvenkar, et al: **Derivation and characterization of two genetically unique human embryonic stem cell lines on in-house-derived human feeders.** *Stem Cells Dev* 2009, **18:**67–77.
5. Kannagi R, Cochran NA, Ishigami F, Hakomori S, Andrews PW, Knowles BB and Solter D: **Stage-specific embryonic antigens (SSEA-3 and SSEA-4) are epitopes of a unique globo-series ganglioside isolated from human teratocarcinoma cells*.*** *EMBO J* 1983, **2:**2355-2361.
6. Gang EJ, Bosnakovski D, Figueiredo CA, Visser JW and Perlingeiro RC: **SSEA-4 identifies mesenchymal stem cells from bone marrow.** *Blood* 2007, **109:**1743-1751.
7. Wojakowski W, Tendera M, Kucia M, Zuba-Surma E, Paczkowska E, Ciosek J, Haasa M, Król M, Kazmierski M, Buszman P, Ochaa A, Ratajczak J, Machaliski B and Ratajczak MZ: **Mobilization of bone marrow-derived Oct-4+ SSEA-4+ very small embryonic-like stem cells in patients with acute myocardial infarction.** *J Am Coll Cardiol* 2009, **53**:1-9.
8. Rappa G, Fodstad O and Lorico A: **The stem cell-associated antigen CD133 (Prominin-1) is a molecular therapeutic target for metastatic melanoma.** *Stem Cells* 2008, **26:**3008-3017.
9. Vander Griend DJ, Karthaus WL, Dalrymple S, Meeker A, DeMarzo AM and Isaacs JT: **The role of CD133 in normal human prostate stem cells and malignant cancer-initiating cells.** *Cancer Res* 2008, **68:**9703-9711.
10. Tirino V, Camerlingo R, Franco R, Malanga D, La Rocca A, Viglietto G, Rocco G, Pirozzi G: [**The role of CD133 in the identification and characterisation of tumour-initiating cells in non-small-cell lung cancer.**](http://www.ncbi.nlm.nih.gov/pubmed/19464919) *Eur J Cardiothorac Surg* 2009, **36:**446-453.
11. Wu Y and Wu PY: **CD133 as a marker for cancer stem cells: Progresses and Concerns.** *Stem Cells Dev* 2009, **18**:1127-1134.
12. Bhartiya D, Shaikh A, Nagvenkar P, et al.: **Very small embryonic-like stem cells with maximum regenerative potential get discarded during cord blood banking and bone marrow processing for autologous stem cell therapy,** *Stem Cells and Development* 2012 21, (1), 1-6.
13. Lange UC, Adams DJ, Lee C, Barton S, Schneider R, Bradley A and Surani MA: **Normal germ line establishment in mice carrying a deletion of the Ifitm/Fragilis gene family cluster.** *Mol Cell Biol* 2008, **28:**4688-4696.
14. Zou K, Hou L, Sun K, Xie W and Wu J: **Improved efficiency of female germline stem cell purification using fragilis-based magnetic bead sorting.** *Stem Cells Dev* 2011, **20:**2197-2204.
15. Feeley EM, Sims JS, John SP, Chin CR, Pertel T, Chen LM, Gaiha GD, Ryan BJ, Donis RO, Elledge SJ and Brass AL: **IFITM3 inhibits influenza A virus infection by preventing cytosolic entry.** *PLoS Pathog* 2011, **7** e1002337.
16. Bortvin A, Goodheart M, Liao M and Page DC: **Dppa3 / Pgc7 / Stella is a maternal factor and is not required for germ cell specification in mice.** *BMC Dev Biol* 2004, **23:** 2.
17. Payer B, Saitou M, Barton SC, Thresher R, Dixon JP, Zahn D, Colledge WH, Carlton MB, Nakano T and Surani MA: **Stella is a maternal effect gene required for normal development in mice.** *Curr Biol 2003,* **13:**2110-2117.
18. Payer B, Chuva de Sousa Lopes SM, Barton SC, Lee C, Saitou M and Surani MA: **Generation of stella-GFP transgenic mice: a novel tool to study germ cell development.** *Genesis* 2006, **44:**75-83.
19. Cauffman G, Van de Velde H, Liebaers I and Van Steirteghem A: **DAZL expression in human oocytes, preimplantation embryos and embryonic stem cells.** *Mol Hum Reprod* 2005, **11:** 405-411.
20. Collier B, Gorgoni B, Loveridge C, Cooke HJ and Gray NK: **The DAZL family proteins are PABP-binding proteins that regulate translation in germ cells.** *EMBO J* 2005, **24:** 2656-2666.
21. Vitt UA, Mazerbourg S, Klein C and Hsueh AJ: **Bone morphogenetic protein receptor type II is a receptor for growth and differentiation factor-9.** *Biol Reprod* 2002, **67:**473-480.
22. Mazerbourg S and Hsueh AJ: **Growth differentiation factor-9 signaling in the ovary.** *Mol Cell Endocrinol.* 2003, **202**:31-36.
23. Gilchrist RB, Ritter LJ, Myllymaa S, Kaivo-Oja N, Dragovic RA, Hickey TE, Ritvos O and Mottershead DG: **Molecular basis of oocyte-paracrine signalling that promotes granulosa cell proliferation*.*** *J Cell Sci* 2006, **119**:3811-3821.
24. Knight PG and Glister C: **TGF-beta superfamily members and ovarian follicle development.** *Reproduction* 2006, **132:**191-206.
25. Castrillon DH, Quade BJ, Wang TY, Quigley C and Crum CP: **The human VASA gene is specifically expressed in the germ cell lineage.** *Proc Natl Acad Sci U S A* 2000, **97:**9585-9590.
26. Raz E: **The function and regulation of vasa-like genes in germ-cell development.** *Genome Biol 2000,* **1** REVIEWS 1017.
27. Zeeman AM, Stoop H, Boter M, Gillis AJ, Castrillon DH, Oosterhuis JW and Looijenga LH: **VASA is a specific marker for both normal and malignant human germ cells.** *Lab Invest.* 2002, **82:** 159-166.
28. [Bukovsky A](http://www.ncbi.nlm.nih.gov/pubmed?term=Bukovsky%20A%5BAuthor%5D&cauthor=true&cauthor_uid=18256545), [Caudle MR](http://www.ncbi.nlm.nih.gov/pubmed?term=Caudle%20MR%5BAuthor%5D&cauthor=true&cauthor_uid=18256545), [Gupta SK](http://www.ncbi.nlm.nih.gov/pubmed?term=Gupta%20SK%5BAuthor%5D&cauthor=true&cauthor_uid=18256545), [Svetlikova M](http://www.ncbi.nlm.nih.gov/pubmed?term=Svetlikova%20M%5BAuthor%5D&cauthor=true&cauthor_uid=18256545), [Selleck-White R](http://www.ncbi.nlm.nih.gov/pubmed?term=Selleck-White%20R%5BAuthor%5D&cauthor=true&cauthor_uid=18256545), [Ayala AM](http://www.ncbi.nlm.nih.gov/pubmed?term=Ayala%20AM%5BAuthor%5D&cauthor=true&cauthor_uid=18256545), [Dominguez R](http://www.ncbi.nlm.nih.gov/pubmed?term=Dominguez%20R%5BAuthor%5D&cauthor=true&cauthor_uid=18256545): **Mammalian neo-oogenesis and expression of meiosis-specific protein SCP3 in adult human and monkey ovaries.** [*Cell Cycle*](http://www.ncbi.nlm.nih.gov/pubmed/18256545) 2008, **7:**683-686.
29. Basu B, Desai R, Balaji J, Chaerkady R, Sriram V, Maiti S and Panicker MM: **Serotonin in pre-implantation mouse embryos is localized to the mitochondria and can modulate mitochondrial potential.** *Reproduction* 2008, **135**: 657–669.
30. Neupert W: **Protein import into mitochondria.** *Ann Rev Biochem* 1997, **66:**863-917.
31. Kroemer G, Dallaporta B, Resche-Rigon M: **The mitochondrial death/life regulator in apoptosis and necrosis.** *Annu. Rev. Physiol* 1998, **60:** 619-642.
32. Tafani M, Karpinich NO, Hurster KA, Pastorino JG, Schneider T, Russo MA, Farber JL: **Cytochrome c release upon Fas receptor activation depends on translocation of full-length bid and the induction of the mitochondrial permeability transition***. J. Biol. Chem* 2002, **277:**10073–10082.
