# Supplementary figures and images for: Dynamics associated with spontaneous differentiation of ovarian stem cells in vitro
Source: J Ovarian Res. 2014 Feb 25;7:25. doi: 10.1186/1757-2215-7-25 (PMC4234975; doi:10.1186/1757-2215-7-25)

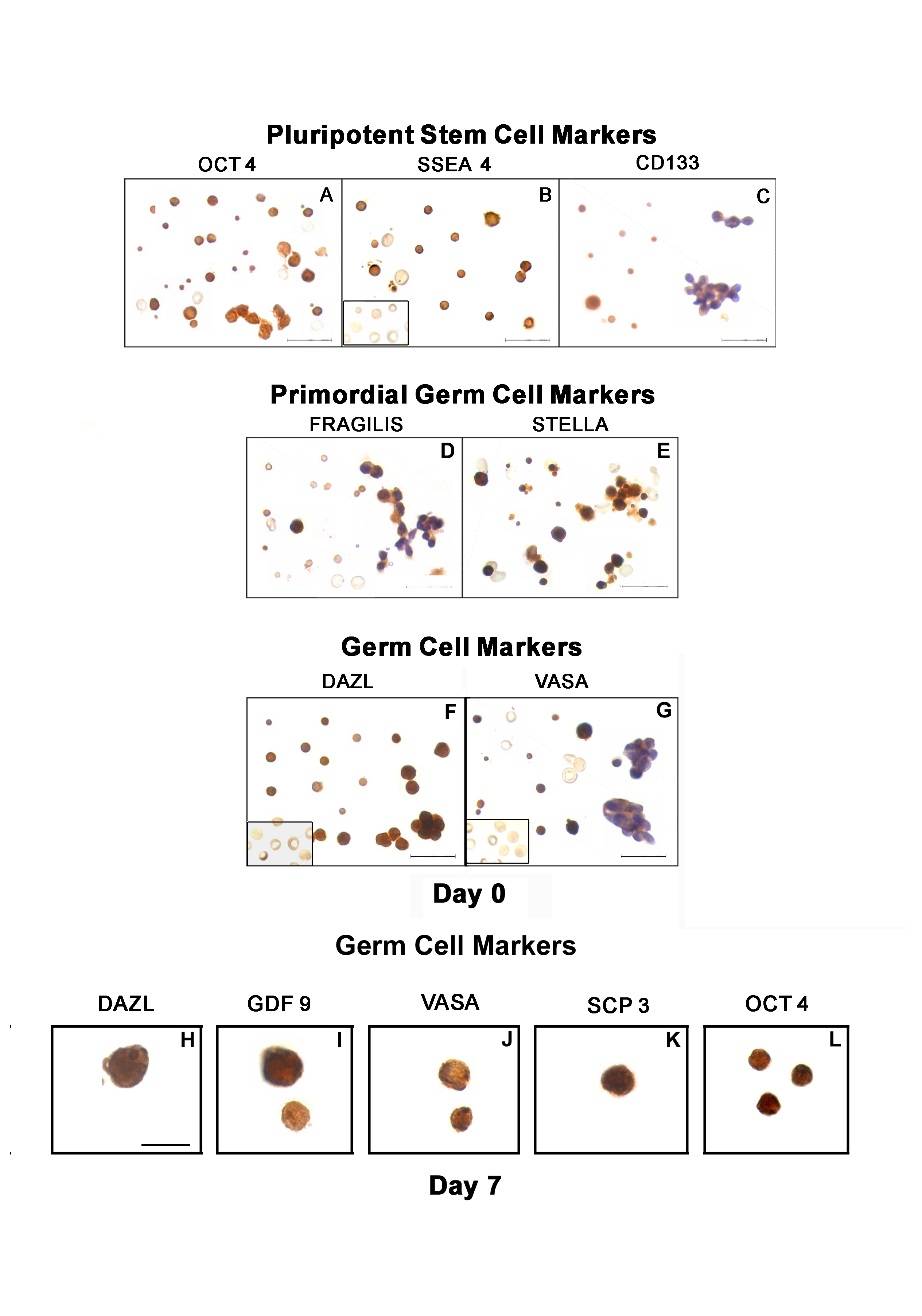

Supplement: Additional file 2: Figure S1 — Immuno-characterization of ovarian stem cells on Day 0 and Day 7. [file 1757-2215-7-25-S2.jpeg]

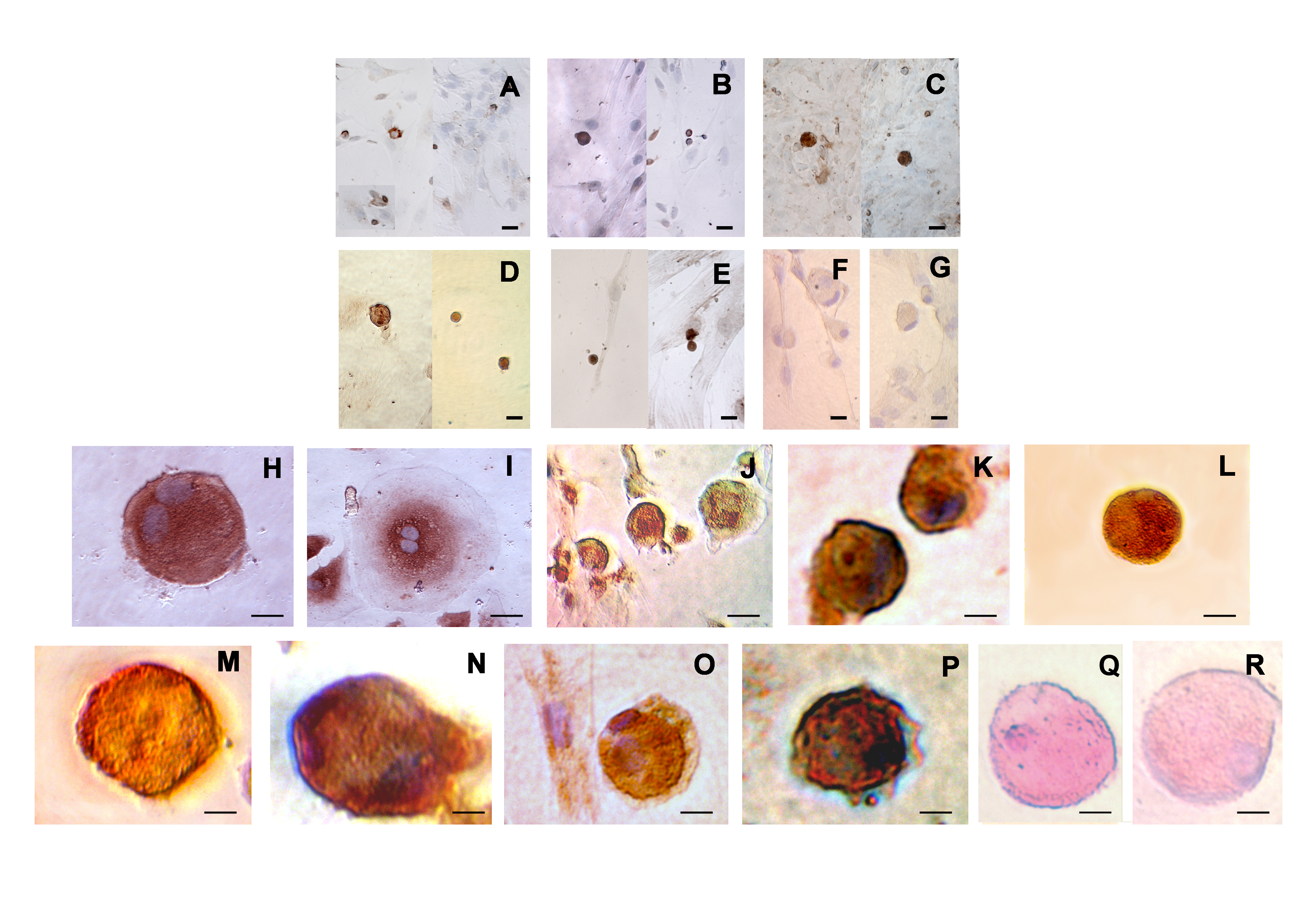

Supplement: Additional file 3: Figure S2 — Immuno-characterization of germ cells in OSE cultures after three weeks. [file 1757-2215-7-25-S3.tiff]

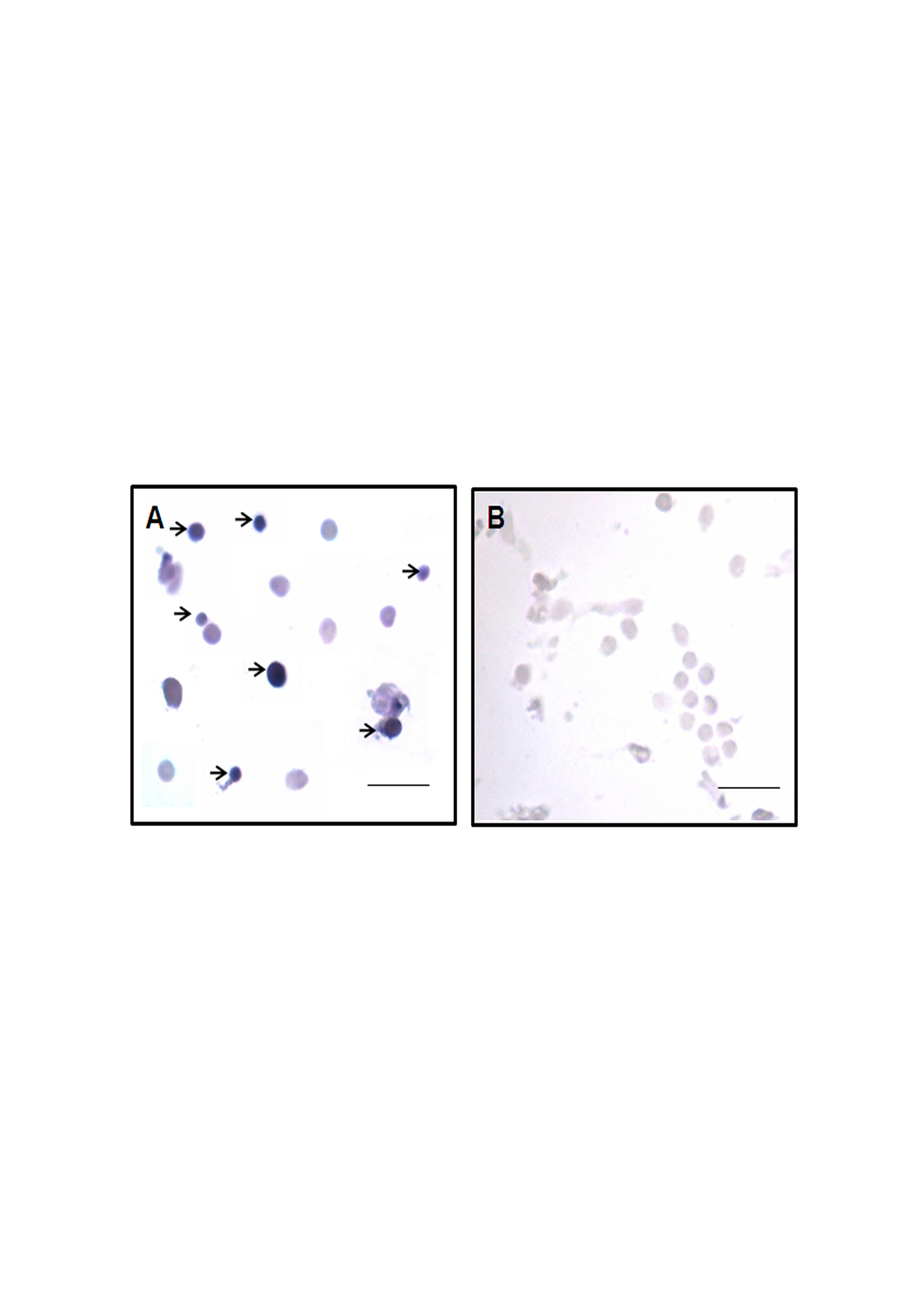

Supplement: Additional file 4: Figure S3 — Localization of Oct-4 mRNA in sheep ovarian stem cells. [file 1757-2215-7-25-S4.tiff]

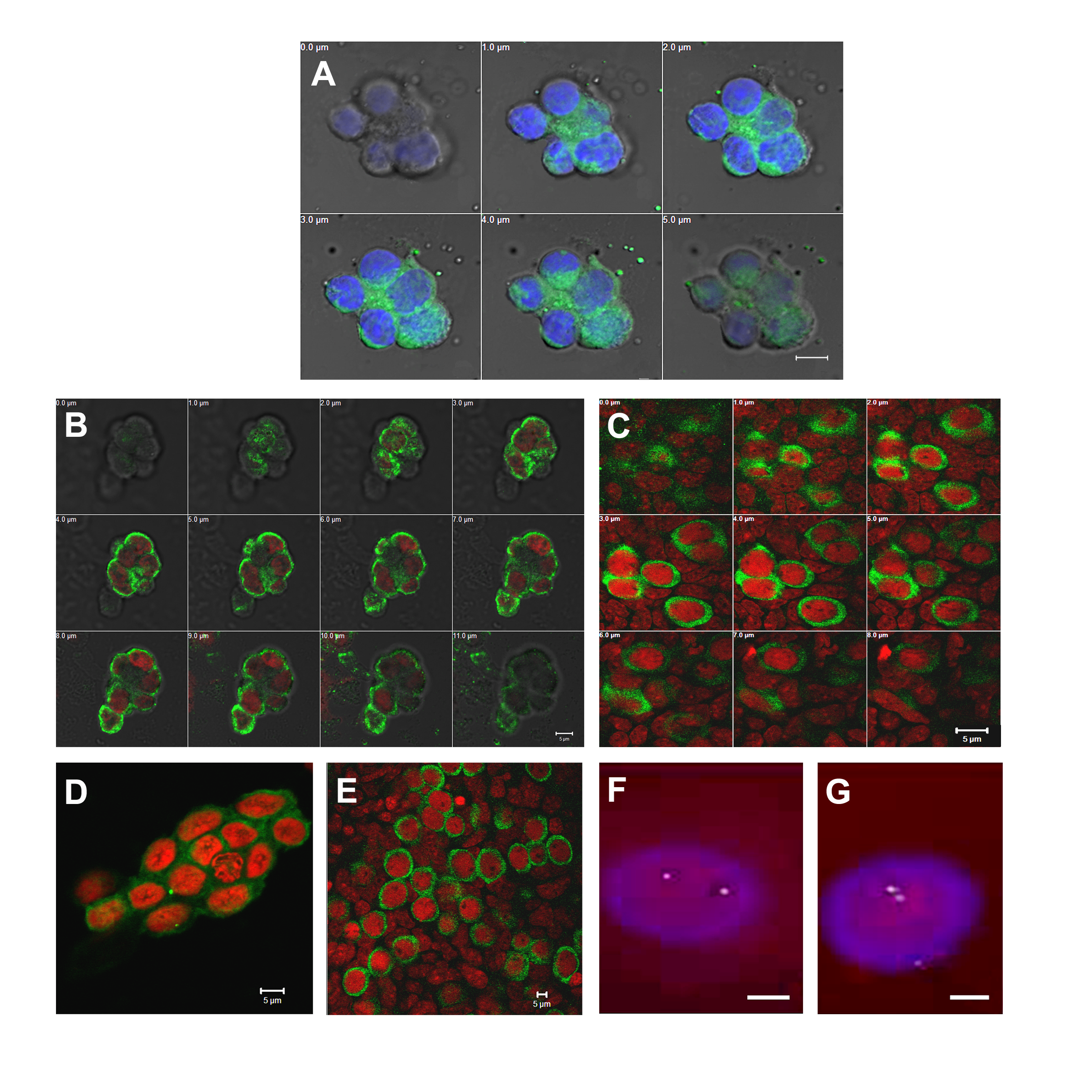

Supplement: Additional file 7: Figure S4 — Confocal z-stack composites of germ cell nests in OSE smears. [file 1757-2215-7-25-S7.tiff]
